# Large Buffering Effect of the Duodenal Bulb in Duodenal Switch: a Wireless pH-Metric Study

**DOI:** 10.1007/s11695-017-2574-0

**Published:** 2017-02-07

**Authors:** Z Bekhali, J Hedberg, H Hedenström, M Sundbom

**Affiliations:** 1 Department of Surgical Science, Upper Gastrointestinal Surgery, Uppsala University, SE-751 85 Uppsala, Sweden; 2 Gävle county Hospital, 801 88 Gävle, Sweden; 3 Department of Medical Science, Clinical Physiology, Uppsala University, SE-751 85 Uppsala, Sweden

**Keywords:** Bariatric surgery, Duodenal switch, Marginal ulcer, pH-metry

## Abstract

**Introduction:**

Bariatric procedures result in massive weight loss, however, not without side effects. Gastric acid is known to cause marginal ulcers, situated in the small bowel just distal to the upper anastomosis. We have used the wireless BRAVO™ system to study the buffering effect of the duodenal bulb in duodenal switch (DS), a procedure in which the gastric sleeve produces a substantial amount of acid.

**Methods:**

We placed a pre- and a postpyloric pH capsule in 15 DS-patients (seven men, 44 years, BMI 33) under endoscopic guidance and verified the correct location by fluoroscopy. Patients were asked to eat and drink at their leisure, and to register their meals for the next 24 h.

**Results:**

All capsules but one could be successfully placed, without complications. Total registration time was 17.2 (1.3–24) hours prepyloric and 23.1 (1.2–24) hours postpyloric, with a corresponding pH of 2.66 (1.74–5.81) and 5.79 (4.75–7.58), *p* < 0.01. The difference in pH between the two locations was reduced from 3.55 before meals to 1.82 during meals, *p* < 0.01. Percentage of time with pH < 4 was 70.0 (19.9–92.0) and 13.0 (0.0–34.6) pre and postpylorically, demonstrating a large buffering effect.

**Conclusion:**

By this wireless pH-metric technique, we could demonstrate that the duodenal bulb had a large buffering effect, thus counteracting the large amount of gastric acid passing into the small bowel after duodenal switch. This physiologic effect could explain the low incidence of stomal ulcers.

## Introduction

Compared to conservative methods, bariatric surgery provides sustainable weight loss, high resolution of comorbidities and decreased overall mortality [1]. The development of bariatric surgery has been experimental, and today several different procedures are used. Roux-en-Y gastric bypass (RYGBP) is commonly performed and considered gold standard by many authors [2]. Another procedure, duodenal switch (DS), is used, preferably in patients with super obesity (body mass index, BMI, over 50 kg/m^2^), and yields remarkable weight loss in this group of patients [3, 4]. DS consists of two different parts. First, a gastric tube is created by preforming a vertical sleeve gastrectomy to reduce the volume of ingested food. The duodenal bulb is divided two to 4 cm distal to the pylorus and anastomosed to the last 2.5-m of distal ileum (alimentary limb). Second, the remaining small bowel, carrying bile and pancreatic juice, is anastomosed 1 m from the ileocecal valve, resulting in decreased uptake of ingested nutrients.

All bariatric procedures have side effects because of major changes in gastrointestinal physiology. The connection of stomach and its acid-producing mucosa to the small bowel can result in marginal ulcers, an ulcer situated just distal to the anastomosis. The incidence of marginal ulcer ranges between 0.6 and 16% after RYGBP [5, 6], while it is substantially less frequent in patients having had DS around 0.3% [4]. As the gastric sleeve in DS contains a much larger amount of acid-producing gastric mucosa than the small gastric pouch in RYGBP, the differences in marginal ulcer incidence could be viewed as paradoxical. The role of included duodenal bulb in this has not been investigated.

Different pH-measuring systems exist, for instance the classic catheter-based technique and the wireless BRAVO™ capsule. The BRAVO™ system (Given Imaging, Yokneam, Israel), developed to measure pH in gastro-esophageal reflux disease, has the advantage of longer registration time [7–10], without disturbing the patient’s daily habits. Moreover, intragastric and duodenal pH monitoring has been implemented with BRAVO™ system with good reliability and promising results [11–17]. We have previous experience in measuring pH in the jejunum, just below the level of gastrojejunal anastomosis in RYGBP, where pH was below four in 10.5 (0.3–37.7) percent of the time during more than 24 h of continuous registration [18].

The aim of the present study was to study the acidity above and below the duodenoileostomy in DS by measuring pre- and postpyloric pH during 24 h of normal activity.

## Materials and Methods

Fifteen patients (seven men, 44 (25–56) years, BMI 33 (25–41) kg/m^2^) who had undergone DS more than 1 year earlier were recruited at our center. All patients were free of abdominal symptoms and medication, except one patient in whom PPI-treatment for gastro-esophageal reflux was discontinued 2 weeks before the study. The study was performed at our endoscopic unit in November 2011–February 2014. A written consent was obtained from all participants.

**Fig. 1:**
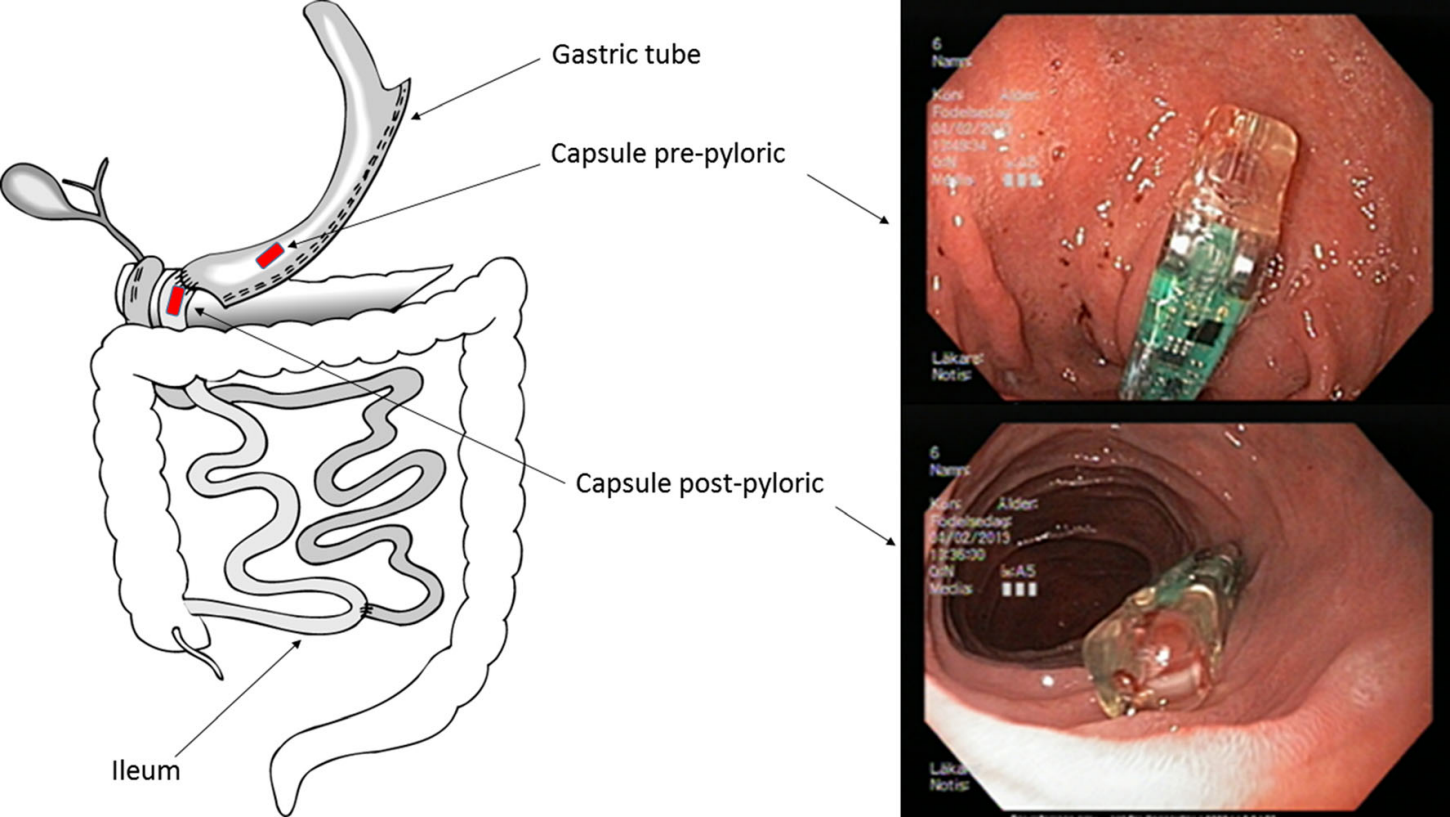
Schematic drawing of duodenal switch (DS) with marked positions for the two Bravo capsules. Endoscopic images representing capsules in place at the pre and postpyloric site

**Fig. 2:**
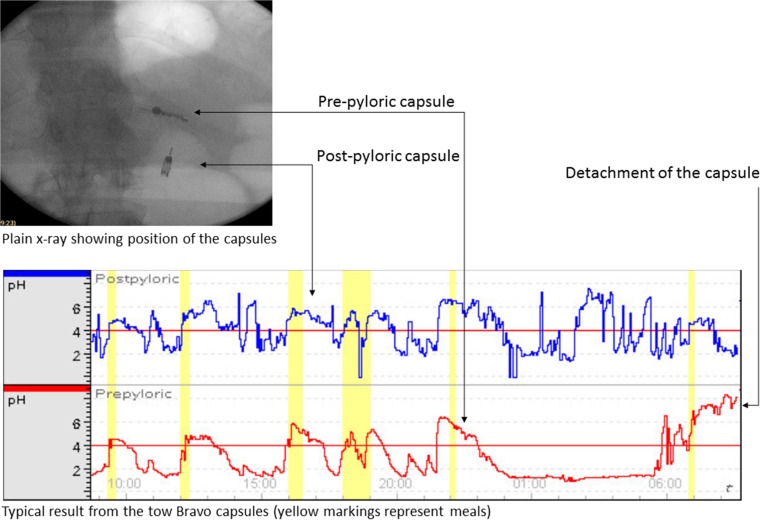
Confirmation by fluoroscopy after placement of the two Bravo capsules and a typical pH curve

After a fasting period of at least 6 h, a cannula was put in the patient’s right arm and a blood sample taken for *Helicobacter pylori* and f- serum gastrin. Prior to upper endoscopy, two wireless BRAVO™ capsules were calibrated and diazepam offered as sedative. With the patient lying on the left side, the delivery system of the first capsule was inserted transorally and followed by the endoscope, to allow direct visual control. To enhance passage through the pylorus, the soft tip of delivery system was grasped by an endoscopic snare. The capsule was placed 1–2 cm below the duodenoileostomy and fastened to the mucosa according to the manufacturer’s instructions, i.e., activating the locking pin after 1 min of suction with 550 mmHg, (Fig.1). After removing the delivery system and endoscope, the prepyloric capsule was placed in the same way, 5 cm oral to pylorus. The correct placement of the capsules was confirmed by fluoroscopy with patient lying in the supine position (Fig.2). The wireless pH recording was activated and patients were asked to carry the portable receiver in a band over the shoulder. Patients were also asked to press a specific button on the receiver whenever ingesting something and to record the meal in a paper protocol. During the 24-h registration, patients were encouraged to eat at their leisure. The location of the capsules was verified by repeated fluoroscopy when the patient returned the recording device on the following day.

pH data was uploaded to the Polygram NET version 4.2 (Given Imaging, Yokneam, Israel). Sudden change in the pH curve with consistently neutral pattern or loss of signal indicated the detachment of the capsule (Fig.2). The total registration time before the detachment of the capsule, median pH during the total registration time, percentage of time with pH < 4, as well as pH 30 min immediately before and during meals, were registered. The difference in mean pH before and during meals was then calculated. All measurements were performed individually by two examiners, and discussed if any inconsequence occurred.

### Statistics

Results are presented as median and range, unless otherwise specified. Mean pH pre and postpylorically was calculated during the whole time of registration for each patient. Time of registration, pH, mean percentage of time with pH < 4 and difference in pH before and during meals were compared using Wilcoxon matched-pair signed rank test. A *p* value <0.05 was considered significant. Statistical analyses were performed by using GraphPad Prism 5.0f (GraphPad Software, Inc., USA).

## Results

All capsules, except one postpyloric capsule, could be successfully placed and the correct location was verified by fluoroscopy. No complications occurred, nor were any complications reported during the pH-registration.

**Fig. 3:**
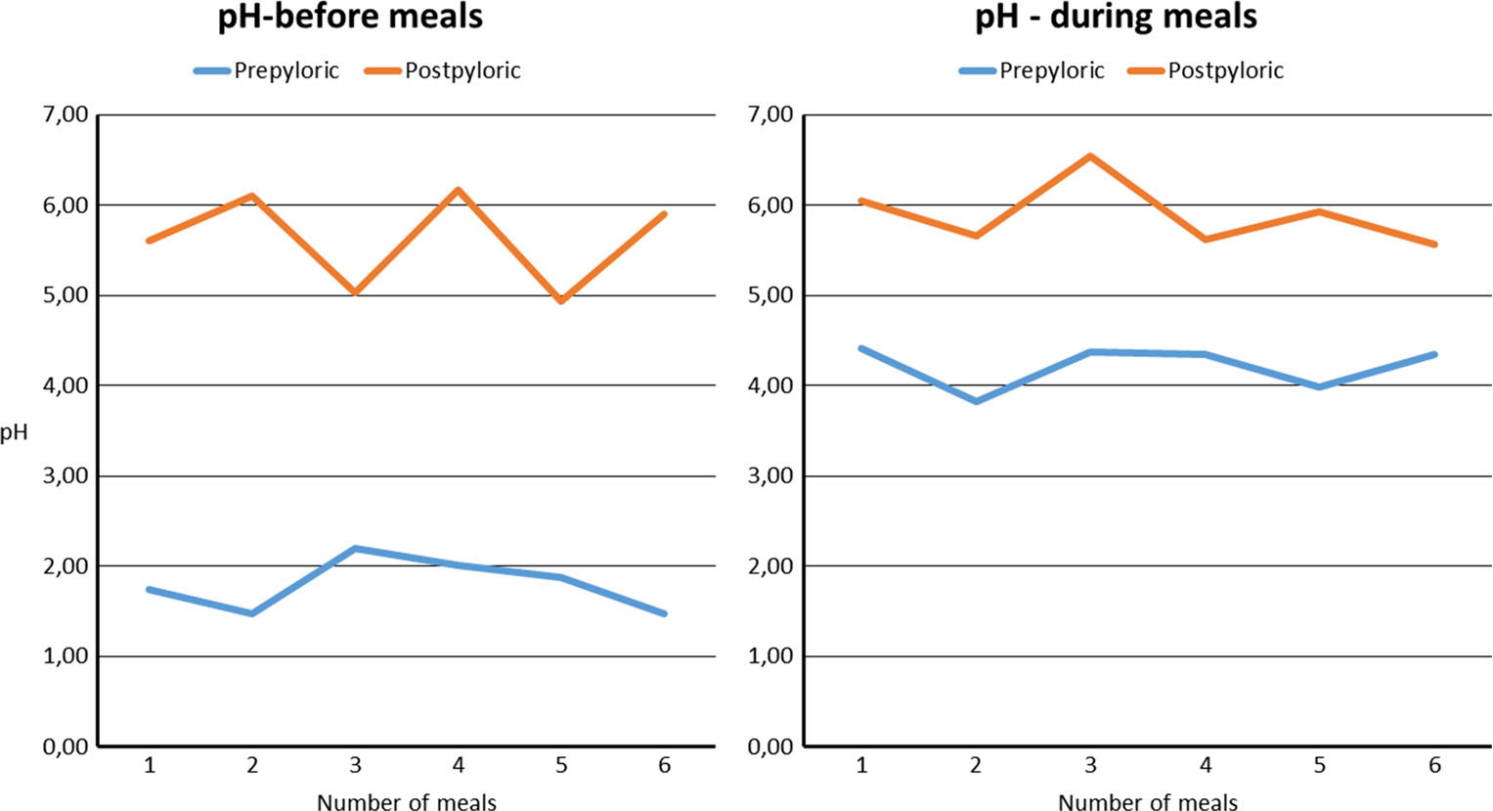
Difference in pre- and postpyloric pH before and during six meals

There was no significant difference in registration time pre- and postpylorically (17.2 vs. 23.1, *p* = 0.36). A lower pH was seen in the gastric tube compared to the postpyloric site, 2.66 (1.74–5.81) vs. 5.79 (4.75–7.58), *p* < 0.01. When calculating percentage of time with pH < 4, the same difference in acidity was apparent, 70.0% (19.9–92.0) and 13.0% (0.0–34.6), respectively, *p* < 0.01. The difference in pH between the two locations was reduced from 3.55 before meals to 1.82 during meals, *p* < 0.01 (Table 1, Fig. 3).

**Table 1:** Registration time and pH data, presented in median (range) and SD, in the 15 studied DS patients

	Median (range)	SD	*p* value
Registration time (*h*)			
Prepyloric	17.2 (1.3–24)	7.3	
Postpyloric	23.1 (1.2–24)	7.8	0.36
pH			
Prepyloric	2.66 (1.74–5.81)	1.1	
Postpyloric	5.79 (4.75–7.58)	0.9	<0.01
Percent of time with pH < 4			
Prepyloric	70.0 (19.9–92.0)	19.9	
Postpyloric	13.0 (0.0–34.6)	12.3	<0.01
Difference in pH			
Before meals	3.55 (1.60–4.85)	0.8	
During meals	1.82 (0.36–3.64)	0.8	<0.01

Wilcoxon matched-pair signed rank test was used for all comparisons

Serology for *H pylori* was negative in all patients. Serum gastrin varied between <10 and 28 pmol/L (reference interval <55), and was thus within normal interval in all patients. No correlation was seen to the measured pH levels.

## Discussion

In this pH-metric study, we could demonstrate that the duodenal bulb has a large buffering effect. Physiologically, this seems to counteract the large amount of acid-producing mucosa in DS. As expected, the difference between pre- and postpyloric pH was reduced during meals, when ingested food increased the gastric pH.

Although conventional placement of the BRAVO™-capsule is done after removal of the endoscope, we used direct endoscopic guidance to place the two capsules. Direct placement avoids a repeated endoscopy to confirm the placement of the capsule and is associated with shorter procedure time and less discomfort for the patients [8, 12, 19]. An acute angulation of the gastric tube imposed some difficulties in advancing the capsule distally through the pylorus, and the use of an endoscopic snare was very helpful. The repeated fluoroscopic examinations, directly after placing the capsule and on the following morning, verified the correct placement of the capsules.

Using only suction and the locking pin, we achieved full 24-h registration for four of the prepyloric capsules and seven postpyloric, corresponding to 26 and 50%, respectively. Registration time is reported to be increased by securing the capsule onto the gastric wall by placing an endoscopic hemoclip on top of a thread tied to the capsule [11, 13, 14]. In our case, registration time was anyhow limited to 24 h, by allowing all data from the two parallel BRAVO™-capsules to be recorded on one receiver. When using separate receivers for each capsule, registration times of up to 48 h onto the gastric wall has been demonstrated [12], as well as up to 4 days in the esophagus with two receivers consecutively calibrated to the same capsule [20].

In our previous measurements, just below the gastrojejunal anastomosis, in 21 RYGBP-patients, median percentage of time with pH < 4 was 10.5% [18], compared to 13.0% in the present study. Although the large gastric remnant in DS results in high acidity (68.7% of the time with pH < 4), the alkaline mucus produced by the Brunner’s glands, located in the first few centimeters of the duodenum, manages to keep the pH in the small bowel on almost the same level as in RYGBP. The high amount of excreted bicarbonate ions will immediately neutralize of the majority of all gastric acid passing into the duodenum. Moreover, the mucus is known to have a protective effect against the erosive acidic gastric content on the underlying endothelium [21, 22]. Theoretically, bile reflux could also contribute to a reduced ulcer incidence, but in DS this requires a retrograde flow through 150 cm of the alimentary limb, which seems unlikely. Furthermore, DS has been described as an operation of choice for pathologic transpyloric duodenogastric reflux [23]. Finally, the ileal mucosa might be more resilient to acid exposure compared to the jejunum used in RYGBP, although these mechanisms remain to be elucidated.

Intragastric pH is affected by different factors including meals, day and night time, medication, vagotomy, presence of *H pylori*, duodenogastric reflux, and gastrin levels [24] etc. These physiological variations have studied by both conventional pH measurements and measurements by the Bravo system [12, 25–27]. During fasting, pH is <4 most of the time in a healthy stomach. Ingestion of food increases the intragastric pH as a result of the buffering effect, usually returning to baseline after 2 h. We could verify the change in intragastric pH during meals, and in addition, we could demonstrate an increase in postpyloric pH, probably occurring due to increased buffering of ingested food passing into the duodenal bulb.

The repeated fluoroscopic examinations, directly after placing the capsule and on the following morning, verifying the exact location, are among the strengths of the present study. The possibility for patients to continue with their ordinary life, without having nasogastric catheters, and the possibility to perform detailed measurements, for example before and after meals have been favorable with the present technique. We did not achieve full 24-h registrations in all patients, which could be regarded as a weakness, especially since four patients had to be excluded from the analysis regarding pH difference before and during meals.

In conclusion, we could demonstrate that the duodenal bulb has a large buffering effect, thus counteracting the large amount of gastric acid passing into the small bowel after duodenal switch. This physiologic effect could contribute to the low incidence of marginal ulcers.
